# Dietary 0.10% chlorogenic acid improves the meat quality of Shaziling pigs

**DOI:** 10.3389/fvets.2026.1846532

**Published:** 2026-07-31

**Authors:** Wenjie Luo, Zihao Fan, Yu Liao, Jing Zhou, Xin Wang, Chaoyue Wen, Qiuping Guo, Yehui Duan, Nan Hu

**Affiliations:** 1 Hunan University of Medicine, School of Medical Informatics and Engineering, Huaihua, Hunan, China; 2 College of Food Science and Technology, Hunan Agricultural University, Changsha, Hunan, China; 3 Hunan Provincial Key Laboratory of Animal Nutritional Physiology and Metabolic Process, National Engineering Laboratory for Pollution Control and Waste Utilization in Livestock and Poultry Production, Institute of Subtropical Agriculture, Chinese Academy of Sciences, Changsha, Hunan, China

**Keywords:** chlorogenic acid, fatty scids, meat quality, metabolic profile, Shaziling pigs

## Abstract

**Introduction:**

This study aimed to explore the effects of chlorogenic acid (CGA) on the meat quality and metabolomic profiling of Shaziling pigs.

**Methods:**

In a 51-day experiment, 48 healthy male Shaziling pigs were randomly divided into two groups: a control (Con) group (basic diets) and a CGA-treated group (basic diets + 0.10% CGA).

**Results:**

The results showed that compared to the Con group, CGA supplementation tended to increase the final weight and dressing percentage of Shaziling pigs. Moreover, CGA increased the intramuscular fat (IMF) content and the pH24h value and decreased the drop loss. CGA also increased the concentrations of inosine-5′-monophosphate (IMP), glutamate, C18:1n9, and MUFAs, and decreased the content of C18:2n6, n6 PUFAs, PUFAs, n6/n3 PUFA ratio in the *longissimus dorsi dorsimuscle*. CGA supplementation increased the content of muscular 4-hydroxy hexenal, cinobufagin, and NAD, and decreased the content of muscular lactic acid, linoleic acid and stearidonic acid. Correlation analyses showed that these increased metabolites were positively associated with the IMP content and/or negatively associated with the drop loss, and these decreased metabolites follow the opposite pattern.

**Discussion:**

In conclusion, these results indicated that CGA could improve the meat quality of Shaziling pigs, and the effects might be associated with improved muscular metabolites.

## Graphical abstract

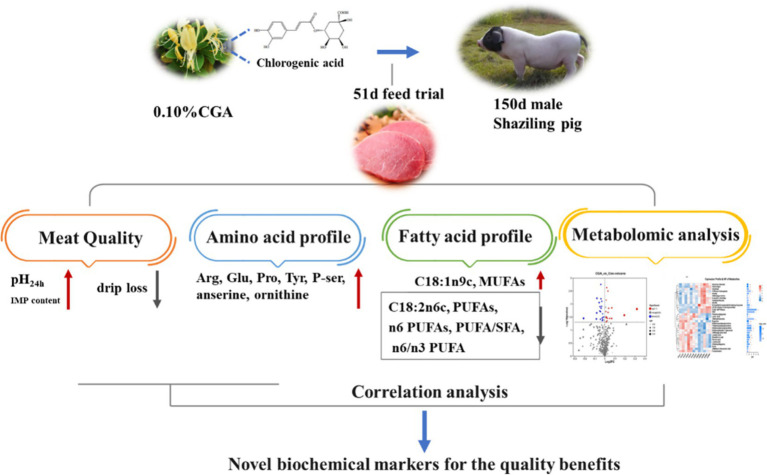

## 1 Introduction

With the improvement of living standards, the demand for high-quality food products is on the rise. Pork, being highly nutritious, is one of the most consumed meats worldwide, and has therefore garnered significant attention (1). China has the world’s most diverse pig breeds, accounting for one-third of the global pig population (2). Shaziling pigs, a prominent indigenous breed from Hunan Province, are favored by consumers due to their premium meat quality and attractive flavor (3, 4). In this context, how to effectively balance the development of the swine industry with food safety assurance has become an urgent issue to address.

The development and use of safe, natural, and effective feed additives that replace antibiotics may solve this issue. Chlorogenic acid (CGA) is a naturally water-soluble polyphenol formed from the esterification of trans-cinnamic acid and quinic acid, and is found in a variety of foods and herbs, including coffee beans, artichokes, eucommia, grapes, kiwifruit, honeysuckle, wormwood, and tea (5). CGA has been reported to possess a variety of biological properties, including antioxidant (6), anti-inflammatory (7), antibacterial (8), antiviral (9), hypoglycemic (7), and hypolipidemic activities (10), leading to a wide range of applications in animal production and human health care. Therefore, CGA is regarded as a potential feed additive to improve animal health and meat quality. Our and other studies have examined the application of CGA in finishing pigs in efforts to improve various aspects of meat quality, including color, drip loss, water-holding capacity, and ultimate pH (11–15), and 0.04% CGA is recommended in Large × White × Landrace finishing pigs (13). However, most current studies in this field were conducted on Western commercial pig breeds, which may not be suitable for Chinese indigenous pig breeds, owing to their fundamental genetic, physiological, and metabolic differences. To the best of our knowledge, the effect of dietary CGA supplementation on meat quality of Shaziling pigs has not been reported until now. The present study will fill a gap in this field.

Given that the supreme meat quality of Shaziling pigs is its core market value, any strategy that can further augment its natural quality advantages holds tremendous economic and brand significance. Therefore, this study aimed to investigate the effects of dietary CGA supplementation on the carcass traits, meat quality, muscular profiles of amino acids and fatty acids, and muscular metabolic profiles of Shaziling pigs from 150 to 210 days of age, since our previous studies have indicated that 150–210 days of age was the critical window for changes in muscular amino acids, fatty acids, and metabolites of Shaziling pigs (16). The findings from this research will provide a scientific foundation for employing CGA as a novel nutritional strategy to enhance the value of indigenous pig production, aligning with the principles of green, healthy, and high-quality animal husbandry.

## 2 Materials and methods

### 2.1 Animal ethics statement

All animal procedures in this study were conducted in accordance with the guidelines of the Animal Care Committee of the Institute of Subtropical Agriculture, Chinese Academy of Sciences, and complied with Chinese guidelines on experimental protocols and animal welfare (ISA-2020-023).

### 2.2 Experimental protocols

Forty-eight healthy male Shaziling pigs with an average body weight (BW, 150 days, 31.56 ± 0.95 kg) were assigned to two groups based on body weight, that is, the control (Con) group and CGA group. Each group consisted of six replicates, with four pigs per replicate. Pigs in the Con group were fed a basal diet, pigs in the CGA group were fed a basal diet supplemented with 0.1% CGA (>99.9%, Hunan E. K Herb Co., Ltd). The composition and nutrient levels of the diets are presented in Supplementary Table S1. All Shaziling pigs were raised at room temperature at 25–25 °C in animal house of institute of subtropical agriculture, Chinese Academy of Sciences with free access to diets and water for 51 days.

### 2.3 Growth performance

The initial and final BW of Shaziling pigs (*n =* 48) were weighed individually on an empty stomach before morning feeding on the 1st and 51st day of the trial period, and were recorded as the initial BW and final BW, respectively. The average daily feed intake (ADFI), average daily gain (ADG), and feed conversion rate (FCR) were calculated by recording the BW, feed intake, and leftover feed of pigs (17).

### 2.4 Sample collection

At the end of the trial, one Shaziling pig closest to the average BW of the pigs in each replicate was selected, fasted overnight (12 h), and subsequently slaughtered following standard commercial procedures. Before slaughter, about 10 mL blood samples were collected from the jugular vein and centrifuged (3,000 *g* for 20 min at 4 °C) to collect serum. The serum samples were stored at −20 °C for further analyses. After slaughter, the carcass was split down from the middle after scalding, dehairing, peeling, and eviscerating. Thereafter, the *longissimus dorsi* muscle (LDM, approximately 100 g) from the right side of each carcass was sampled and immediately stored at 4 °C for the measurements of meat quality parameters. Additional LDM samples (200 g) were sliced, individually vacuum-packed, and stored at −80 °C for the analysis of amino acid composition, fatty acid composition, and metabolic profiles.

### 2.5 Carcass traits

The left side of the carcass was weighed and then separated into skeletal muscle and fat. The fat mass and muscle mass were weighed and recorded, allowing for the calculation of lean percentage and fat percentage; the loin-eye area was the cross-sectional area of the LDM between the 6th and 7th ribs of the carcass (18). Carcass dissection was performed by trained personnel. Cut weights were recorded using calibrated scales, and loin eye area was measured with a vernier caliper.

### 2.6 Meat quality

The pH values were determined with a pH meter (pH-STAR, SFK-Technology, Denmark) at 45 min and 24 h post-mortem. Meat color was measured using a chromameter (CR-410, Kinica Minolta Sensing Inc., Osaka, Japan) at two different locations to obtain the L* (Lightness), a* (redness), and b* (yellowness) values. The intramuscular fat (IMF) content was measured using petroleum ether and the Soxhlet Extractor method. The cooking loss, drip loss, and shear force of muscles were determined as previously described (18). The inosine-5′-monophosphate (IMP) content was measured by high-performance liquid chromatography as previously described (12).

### 2.7 Serum biochemical parameters

The concentrations of serum total protein (TP), albumin (ALB), urea nitrogen (UN), triglyceride (TG), total cholesterol (TC), high-density lipoprotein cholesterol (HDL-C), and low-density lipoprotein cholesterol (LDL-C) were analysed by using Cobas c-311 coulter chemistry analyzer (Roche Diagnostics International Ltd., Shanghai, China). Biochemical kits for these parameters were purchased from Roche (Shanghai, China).

### 2.8 Muscular free amino acid profiles

For the analysis of muscular free amino acid concentrations, 0.5 g of freeze-dried LDM samples were homogenized with 25 mL of 0.01 M hydrochloric acid and incubated overnight at 4 °C, and then centrifuged at 10,000 *g* for 15 min to obtain the supernatant. Following filtration through a 0.2-μm filter, the filtrate was labeled with iTRAQ reagents (AA 45/32 kit, Applied Biosystems) per the manufacturer’s instructions and analyzed by HPLC (Agilent 1,260 Infinity II). Total FAA (aspartic acid, serine, glutamic acid, alanine, methionine) and total SAA (aspartic acid, serine, glycine, alanine, histidine, arginine, proline, valine, threonine, lysine) were calculated as the sum of their respective constituent amino acids (19, 20).

### 2.9 Muscular fatty acid composition

Gas chromatographic analysis was performed on 150 mg lyophilized muscle samples using an HP 6890 series instrument (Hewlett Packard) equipped with a DB-23 capillary column (122–2,362; Agilent Technologies Inc.) and a flame ionization detector. The column specifications were 60 m in length, 0.25 mm in internal diameter, and 0.25 μm in film thickness as previously described (21).

### 2.10 Metabolomic analysis of longissimus dorsi muscle

The metabolomic profiling of the LDM was performed according to a previously established protocol (22). The procedure involved taking 50 mg of muscle samples and mixing them with a cold methanol/water solution (70% v/v, 1,000 μL) along with an internal standard, 2-chlorophenylalanine (5 μL, 1 μg/mL). The mixture was homogenized at 30 Hz for 3 min, followed by vortexing for 1 min. Subsequently, the samples were centrifuged at 12,000 *g* for 10 min at 4 °C. The supernatant was then filtered through a 0.22 μm filter membrane and stored at −20 °C overnight. Following the initial centrifugation, 200 μL of the supernatant was transferred to a storage vial and stored at −80 °C for subsequent analysis. For metabolite analysis, an ExionLC AD ultra-performance liquid chromatographysystem coupled with a QTRAP® tandem mass spectrometry (MS) system (Applied Biosystems, Foster City, CA) equipped with an Acquity HSS T3 C18 column (2.1 mm × 100 mm, 1.8 μm; Waters, Milford, MA) was used. The mobile phase for chromatography consisted of 0.1% formic acid in water (solvent A) and acetonitrile (solvent B). The metabolites were eluted in a stepwise manner at a flow rate of 0.4 mL/min, with the following gradient: 0–11 min, 5% A; 11–12 min, 10% A; and 12.1–14 min, 95% A. The column temperature was maintained at 40 °C, with an injection volume of 2 μL. Accurate mass measurements were conducted in both positive and negative ion electrospray modes. The triple quadrupole MS was selected for its capability to perform information-dependent MS/MS spectra acquisition. The acquisition software (Analyst 1.6.3, Sciex) continuously assessed the full scan survey MS data, triggering the collection of MS/MS spectra based on predefined criteria. The electrospray ionization source operation parameters included a source temperature of 500 °C, ion spray voltages of 5,500 V (positive) and −4,500 V (negative), ion source gas I at 55 psi, gas II at 60 psi, and a curtain gas pressure of 25 psi, with the collision gas set to high.

### 2.11 Statistical analysis

The experiment data were preliminarily processed with Excel 2016, then the unpaired *t*-tests were performed by Prism7.04 (GraphPad, LaJolla, CA, USA) and used to compare the differences between groups (Con *vs* CGA), *n =* 6/group, and the test data were expressed as “mean ± standard error.” Significant differences from the *t*-test were marked as * for *p* < 0.05, 0.10 < *p* < 0.05 indicated a trend toward statistical significance. The Pearson correlation analysis were performed between significantly difference metabolites and meat quality parammeters.

## 3 Results

### 3.1 Growth performance and carcass traits

As presented in Tables 1, 2, compared to the Con Group, dietary CGA supplementation tended to increase the final BW (*p* = 0.092) and dressing percentage (*p* = 0.067). Dietary CGA supplementation had no significant effects on ADFI, ADG, FCR, and other carcass traits (*p* > 0.05).

**Table 1:** Effects of dietary CGA supplementation on the growth performance of Shaziling pigs.

Items^1^	Con	CGA	*P*-value
Initial BW, kg	31.63 ± 1.03	32.77 ± 1.15	0.479
Final BW, kg	69.60 ± 1.04	72.60 ± 1.22	0.092
ADFI, kg/d	2.57 ± 0.07	2.48 ± 0.08	0.394
ADG, kg/d	0.75 ± 0.02	0.77 ± 0.03	0.396
FCR	3.48 ± 0.18	3.21 ± 0.20	0.354

^1^BW, body weight; ADFI, average daily feed intake; ADG, average daily gain; FCR, feed conversion ratio; Con, control; CGA, chlorogenic acid. Data were presented as mean ± SEM (*n =* 6).

**Table 2:** Effects of dietary CGA supplementation on the carcass traits of Shaziling pigs.

Items^1^	Con	CGA	*P*-value
Dressing percentage, %	64.41 ± 1.11	67.52 ± 1.02	0.067
Average backfat, mm	42.00 ± 1.26	42.50 ± 0.69	0.731
Loin eye area, mm^2^	1130.29 ± 48.00	1079.04 ± 42.31	0.442
Lean percentage, %	35.52 ± 0.77	34.33 ± 0.96	0.359
Fat percentage, %	30.94 ± 0.67	32.09 ± 1.36	0.468

^1^Con, control; CGA, chlorogenic acid. Data were presented as mean ± SEM (*n =* 6).

### 3.2 Meat quality

As presented in Table 3, compared to the Con group, dietary CGA supplementation significantly increased the pH_24 h_ value (*p* = 0.043) and the IMP content (*p* = 0.05) and decreased the drip loss (*p* = 0.039). However, no significant differences were observed in the L*, a*, b*, pH_45 min_ value, cooking loss, and shear force between the two groups (*p* > 0.05).

**Table 3:** Effects of dietary CGA supplementation on the meat quality of Shaziling pigs.

Items^1^	Con	CGA	*P*-value
L* (lightness)	49.74 ± 2.42	48.70 ± 3.66	0.576
a* (redness)	17.61 ± 0.91	17.74 ± 1.15	0.827
b* (yellowness)	4.27 ± 0.62	3.95 ± 1.02	0.538
pH_45 min_	6.57 ± 0.14	6.50 ± 0.25	0.581
pH_24 h_	5.43 ± 0.09	5.57 ± 0.12^*^	0.043
Drip loss (24 h), %	3.16 ± 0.59	2.20 ± 0.81^*^	0.039
Cooking loss, %	23.68 ± 3.44	25.33 ± 3.78	0.449
Shear force, *N*	57.19 ± 14.33	62.55 ± 12.17	0.519
IMF, %	3.01 ± 0.068	3.82 ± 0.097^*^	<0.001
IMP, μmol/g	3.03 ± 0.336	4.603 ± 0.621^*^	0.050

^1^IMP, inosine-5′-monophosphate; IMF, intramuscular fat; Con, control group; CGA, chlorogenic acid group. Data were presented as mean ± SEM (*n =* 6). ^*^Values with an asterisk mean significant difference compared with the Con group (*p* < 0.05).

### 3.3 Serum biochemical indexes

As presented in Table 4, compared to the Con Group, dietary CGA supplementation significantly increased the concentrations of TP (*p* = 0.011) and ALB (*p* = 0.011), and tended to decrease the UN content (*p* = 0.064). There were no significant differences in TG (*p* = 0.496), TC (*p* = 0.310), HDL-C (*p* = 0.171), and LDL-C (*p* = 0.429) between the two groups.

**Table 4:** Effects of dietary CGA supplementation on serum biochemical indices of the Shaziling pigs.

Items^1^	Con	CGA	*P*-value
TP, g/L	70.44 ± 2.58	85.65 ± 3.78*	0.011
ALB, g/L	49.38 ± 0.89	60.97 ± 3.25*	0.012
UN, mmol/L	4.47 ± 0.26	3.50 ± 0.39	0.064
TG, mmol/L	0.59 ± 0.08	0.69 ± 0.10	0.496
TC, mmol/L	3.57 ± 0.22	3.89 ± 0.20	0.310
LDL-C, mmol/L	2.24 ± 0.16	2.43 ± 0.16	0.429
HDL-C, mmol/L	1.55 ± 0.09	1.74 ± 0.08	0.171

TP, total protein; ALB, albumin; UN, urea nitrogen; TG, triglyceride; TC, total cholesterol; LDL-C, low-density lipoprotein-cholesterol; HDL-C, high-density lipoprotein cholesterol. Con, control; CGA, chlorogenic acid. Data were presented as mean ± SEM (*n =* 6). ^*^Values with an asterisk mean significant difference compared with the Con group (p < 0.05).

### 3.4 Muscular amino acid profile

As illustrated in Table 5, compared to the Con group, CGA supplementation significantly increased the concentration of arginine (Arg, *p* = 0.045), glutamate (Glu, *p* = 0.044), proline (Pro, *p* = 0.035), tyrosine (Tyr, *p* = 0.001), phosphatidylserine (P-ser, *p* = 0.022), anserine (*p* = 0.003), and ornithine (*p* = 0.026) in the LDM. Moreover, CGA supplementation tended to increase the concentrations of serine (Ser, *p* = 0.052) and to decrease the concentrations of aspartate (Asp., *p* = 0.073). Dietary CGA supplementation did not significantly affect the concentrations of other amino acids (*p* > 0.05).

**Table 5:** Effects of dietary CGA supplementation on amino acid profiles in the longissimus dorsi muscle of Shaziling pigs.

Items, ug/g	Con	CGA	*P*-value
EAAs			
Histidine	35.24 ± 4.25	34.97 ± 3.10	0.960
Isoleucine	131.74 ± 12.01	126.35 ± 5.65	0.695
Leucine	198.66 ± 14.86	188.69 ± 9.57	0.585
Lysine	120.65 ± 11.31	158.85 ± 23.62	0.175
Methionine	54.41 ± 7.44	54.41 ± 6.38	0.999
Phenylalanine	70.19 ± 5.97	76.88 ± 7.12	0.488
Threonine	78.30 ± 4.82	89.28 ± 3.95	0.109
Valine	139.92 ± 18.89	154.87 ± 9.44	0.394
Total EAAs	829.11 ± 61.64	884.30 ± 48.53	0.498
NEAAs			
Alanine	585.76 ± 61.87	603.48 ± 48.08	0.826
Arginine	53.46 ± 7.09	94.50 ± 16.04*	0.045
Cysteine	17.77 ± 3.31	19.80 ± 3.72	0.693
Aspartate	52.62 ± 5.22	40.46 ± 3.11	0.073
Glutamate	97.98 ± 7.15	126.53 ± 10.26*	0.044
Glycine	301.39 ± 18.91	306.22 ± 24.40	0.879
Proline	54.04 ± 1.74	84.75 ± 12.47*	0.035
Serine	63.35 ± 5.59	80.21 ± 5.24	0.052
Tyrosine	75.27 ± 3.04	101.32 ± 5.14*	0.001
Total NEAAs	1301.46 ± 98.88	1457.27 ± 87.37	0.265
FAA^1^	505.28 ± 36.85	567.72 ± 36.24	0.231
SAA^2^	1203.50 ± 90.98	1322.79 ± 95.92	0.388
Others			
Phosphatidylserine	33.27 ± 3.65	70.86 ± 13.35*	0.022
Anserine	352.51 ± 39.76	1533.00 ± 84.88*	0.003
Carnosine	17800.71 ± 1734.05	20712.38 ± 1529.20	0.236
Taurine	718.30 ± 87.56	823.16 ± 13.35	0.359
Ornithine	16.63 ± 2.15	26.51 ± 3.12*	0.026

^1^FAA, flavor amino acid (sum of Asp., Glu, Gly, and Arg). ^2^SAA, sweet amino acid (sum of Gly, Ser, Thr, Lys, Ala, and Pro). Con, control; CGA, chlorogenic acid; EAAs, essential amino acids; NEAAs, non- essential amino acids. Data were presented as mean ± SEM (*n =* 6). ^*^Values with an asterisk mean significant difference compared with the Con group (*p* < 0.05).

### 3.5 Muscular fatty acid composition

As shown in Table 6, dietary CGA supplementation significantly increased the concentration of oleic acid (C18:1n9c) (*p* = 0.017) and monounsaturated fatty acids (MUFAs) (*p* = 0.013), and significantly decreased the content of linoleic acid (C18:2n6c) (*p* = 0.005), polyunsaturated fatty acids (PUFAs) (*p* = 0.024), and n6 PUFAs (*p* = 0.021) as well as the ratio of PUFA to SFA (*p* = 0.040) and the ratio of n6 to n3 PUFA (*p* = 0.012) in the LDM.

**Table 6:** Effects of dietary CGA supplementation on the fatty acid composition in the longissimus dorsi muscle of Shaziling pigs.

Items, %	Con	CGA	*P*-value
C16:0	26.78 ± 0.46	26.43 ± 0.43	0.596
C16:1	3.08 ± 0.16	3.33 ± 0.29	0.465
C18:0	15.24 ± 0.24	14.78 ± 0.35	0.299
C18:1n9c	37.49 ± 0.53	40.88 ± 1.05*	0.017
C18:2n6c	11.13 ± 0.26	8.74 ± 0.61*	0.005
C20:0	0.25 ± 0.11	0.24 ± 0.13	0.453
C20:1	0.73 ± 0.06	0.78 ± 0.05	0.545
C18:3n3	0.21 ± 0.01	0.21 ± 0.02	0.873
C20:2	0.31 ± 0.02	0.29 ± 0.02	0.580
C20:3n6	0.29 ± 0.03	0.24 ± 0.04	0.332
C20:4n6	2.76 ± 0.35	2.12 ± 0.46	0.305
SFA^1^	43.76 ± 0.62	43.06 ± 0.56	0.425
MUFA^2^	41.41 ± 0.50	45.11 ± 1.12*	0.013
PUFA^3^	14.69 ± 0.45	11.62 ± 1.06*	0.024
n3-PUFA^4^	0.21 ± 0.01	0.21 ± 0.02	0.873
n6-PUFA^5^	14.17 ± 0.45	11.11 ± 1.03*	0.021
PUFA/SFA	0.34 ± 0.01	0.27 ± 0.03*	0.046
n6:n3 PUFA	69.51 ± 5.13	52.20 ± 2.35*	0.012

^1^SFA (saturated fatty acid) = C14:0 + C16:0 + C17:0 + C18:0 + C20:0. ^2^MUFA (monounsaturated fatty acid) = C16:1 C C18:1n9t C C18:1n9c + C20:1 + C22:1n9. ^3^PUFA (polyunsaturated fatty acids) = C18:2n6c + C18:3n3 + C20:2 + C20:3n6 + C20:4n6. ^4^n3PUFA = C18:3n3. ^5^n6 PUFA = C18:2n6c + C20:3n6 + C20n4n6. Con: control, CGA: chlorogenic acid. Data are presented as mean ± SEM (*n =* 6). ^*^Values with an asterisk mean significant difference compared with the Con group (*p* < 0.05).

### 3.6 Metabolomic analysis of muscles

**Figure 1:**
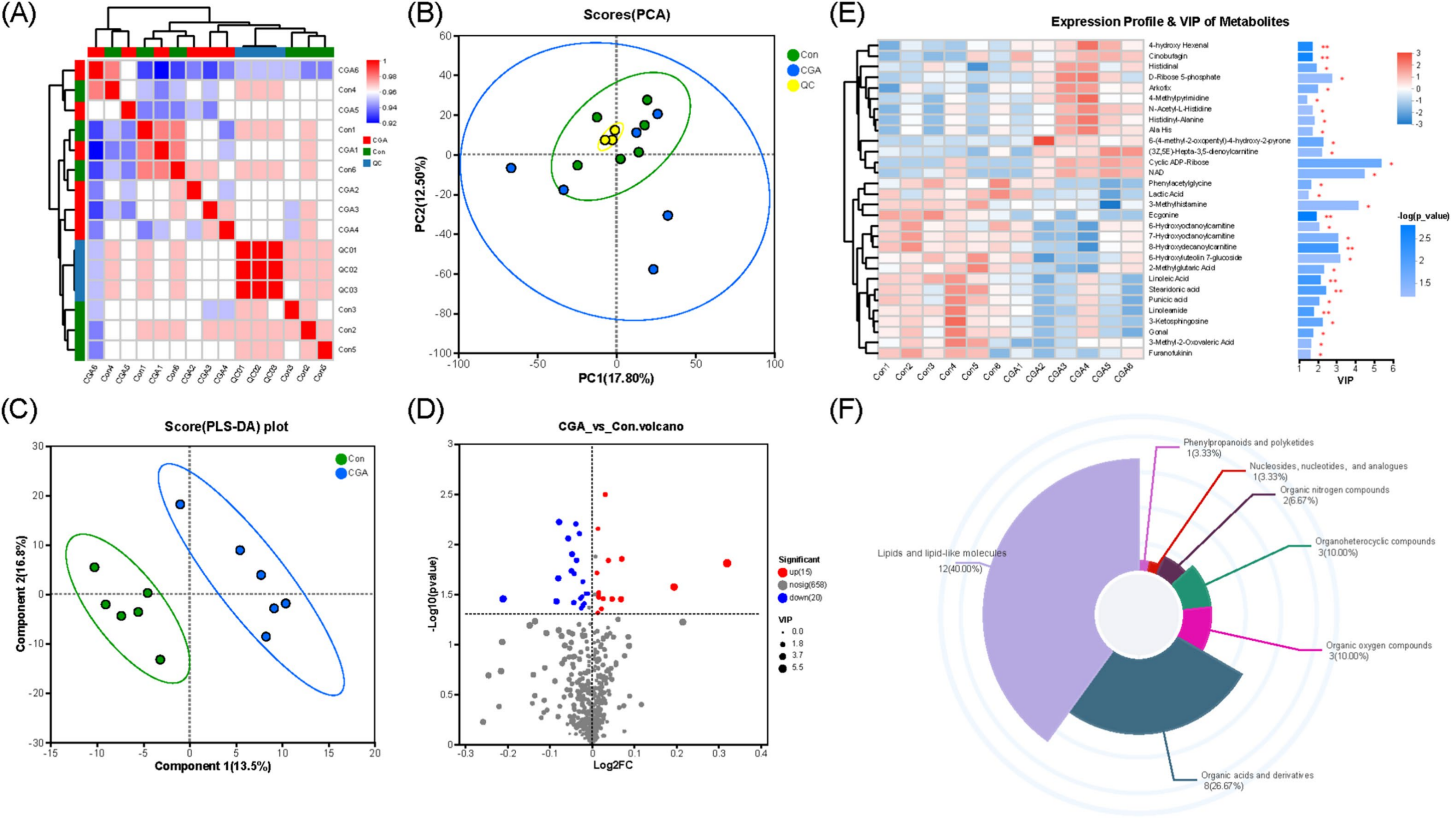
Effects of dietary CGA supplementation on metabolite profiles in the longissimus dorsi muscle of Shaziling pigs. **(A)** Sample correlation heatmap. Pairwise Pearson correlation coefficients between all samples were calculated based on their metabolic profiles and are displayed with a color gradient ranging from blue (low correlation) to red (high correlation). **(B)** Principal component analysis (PCA) score plot. Each point represents an individual sample. **(C)** Partial least squares discriminant analysis (PLS-DA) score plot. **(D)** Volcano plot of differential metabolites between CGA and Con groups. Horizontal dashed lines mark |log10(*p*-value)| ≥ 1. Red dots denote significantly up-regulated metabolites, blue dots indicate significantly down-regulated metabolites, and gray dots represent non-significant changes in CGA. **(E)** Heat-map and two-way hierarchical clustering of the Top-30 differential metabolites (ranked by VIP ≥ 1.0, *p* ≤ 0.05 between CGA and Con groups). Columns represent individual biological replicates (*n =* 6 per group). The left panel shows complete-linkage clustering (Euclidean distance) among metabolites only; the sample order is fixed by individual biological replicates. The right panel displays the corresponding VIP scores shown as a horizontal bar chart (VIP ≥ 1.0). *Indicates a significant difference in metabolite content (*p* ≤ 0.05), while ** denote an extremely significant difference (*p* ≤ 0.01). **(F)** HMDB compound metabolites – Donut Chart. The circular percentage plot displays the relative abundance of metabolite classes annotated by HMDB. Each colored segment represents a distinct chemical category, with percentages calculated from the total number of human metabolome database-confirmed compounds.

Table 7 presents details of the top 25 differentially abundant metabolites, including HMDB identifiers, retention times, match scores, and identification confidence levels. Figure 1A shows a sample correlation heatmap. Figure 1B presents a PCA plot, with PC1 accounting for 17.8% and PC2 for 12.5% of the total variance. Figure 1C illustrates a PLS-DA plot, demonstrating clear discrimination between the two groups. As shown in Figure 1A, the results exhibited distinct clustering in terms ofthe proteome metabolomics profiles between the two groups. Among the identified proteinsmetabolites, 35 differential metabolites were screened out with a fold change >1 and a *p*-value <0.05, of which 15 metabolites were increased and 20 metabolites were decreased in the CGA group. Then, to further investigate the differential metabolites with biological activities in each classification, a heat map was plotted for the Con vs. 0.10% CGA (Figures 1D,E). Compared to the Con group, 0.10% CGA supplementation elevated 12 differential metabolites and reduced 17 differential metabolites in the muscle of Shaziling pigs (VIP value *>* 1). The 12 elevated metabolites were cyclic ADP-ribose, NAD^+^, D-Ribose 5-phosphate, 6-(4-methyl-2-oxopentyl)-4-hydroxy-2-pyrone, (3Z,5E)-Hepta-3,5-dienolycarnitine, histidinyl-alanine, histidinal, Ala-His, N-Acetyl-L-histidine, 4-Methylpyrimidine, Arkofix, cinobufagin, which were predominantly amino acids, peptides, and analogs, and energy metabolism-related substances. The 17 reduced metabolites were lactic acid, 3-methylhistamine, 6-hydroxyluteolin 7-glucoside, 7-hydroxyoctanoylcarnitine, 8-hydroxydecanoylcarnitine, Stearidonic acid, 2-methylglutaric acid, 3-ketosphingosine, linoleic acid, punicic acid, 6-hydroxyoctanoylcarnitine, ecgonine, linoleamide, 3-methyl-2-oxovaleric acid, gonal, phenylacetylglycine, and furanofukinin. Figure 1F is a rose petal diagram showing the superclass distribution of these 30 metabolites: lipids and lipid-like molecules constituted the largest proportion (12, 40.00%), followed by organic acids and derivatives (8, 26.67%), organoheterocyclic compounds (3, 10.00%), organic oxygen compounds (3, 10.00%), organic nitrogen compounds (2, 6.67%), phenylpropanoids and polyketides (1, 3.33%), and nucleosides, nucleotides, and analogues (1, 3.33%).

**Table 7:** Effects of dietary CGA supplementation on the metabolite profiles in the longissimus dorsi muscle of Shaziling pigs.

Metabolite	Formula	Retention time	Library ID	M/Z	Theoretical fragmentation score	Fragmentation score	Mode
Cyclic ADP-Ribose	C15H21N5O13P2	1.1352	HMDB0249529	540.054	0	84	neg
NAD+	C21H27N7O14P2	1.1509	HMDB0000902	662.1026	0	94.7	neg
D-Ribose 5-phosphate	C5H11O8P	0.7185	HMDB0001548	211.001	59.5	0	neg
6-(4-methyl-2-oxopentyl)-4-hydroxy-2-pyrone	C11H13O4-	2.7595	HMDB0304223	227.1141	38.2	0	pos
(3Z,5E)-Hepta-3,5-dienoylcarnitine	C14H23NO4	3.7456	HMDB0241692	302.1961	37.8	0	pos
Histidinyl-Alanine	C9H14N4O3	0.6203	HMDB0304819; HMDB0028878	209.1034	57.5	0	pos
Histidinal	C6H9N3O	0.8663	HMDB0012234	122.0715	63.5	0	pos
Ala His	C9H14N4O3	0.6277	-	227.1138	0	64.6	pos
N-Acetyl-L-Histidine	C8H11N3O3	0.6277	HMDB0032055	198.0874	0	88.7	pos
4-Methylpyrimidine	C5H6N2	0.6203	-	95.06083	0	45.4	pos
Arkofix	C5H10N2O5	0.7753	HMDB0248602	211.0907	48.4	0	pos
Cinobufagin	C26H34O6	6.3241	HMDB0250266	460.2693	66.7	0	pos
Lactic Acid	C3H6O3	0.885	HMDB0001311; HMDB0000190; HMDB0000197	269.0878	0	53.4	neg
3-Methylhistamine	C6H11N3	0.5519	HMDB0001861; HMDB0000898	126.1028	0	40.4	pos
6-Hydroxyluteolin 7-glucoside	C21H20O12	0.7185	HMDB0302732	509.0927	48.8	0	neg
7-Hydroxyoctanoylcarnitine	C15H29NO5	4.7112	HMDB0241695	304.2118	42.2	0	pos
8-Hydroxydecanoylcarnitine	C17H33NO5	5.9612	HMDB0241060	332.243	51.8	0	pos
Stearidonic acid	C18H28O2	6.4166	HMDB0006547	294.2427	73	0	pos
2-Methylglutaric Acid	C6H10O4	1.9324	HMDB0000422	145.0499	0	55.8	neg
3-Ketosphingosine	C18H35NO2	6.4629	HMDB0245914	280.2634	58.1	0	pos
Linoleic Acid	C18H32O2	6.5554	HMDB0000673	298.2739	0	64	pos
Punicic acid	C18H30O2	6.5014	HMDB0030963	296.2583	53.8	0	pos
6-Hydroxyoctanoylcarnitine	C15H29NO5	4.9172	HMDB0241741	304.2118	38	0	pos
Ecgonine	C9H15NO3	4.0268	HMDB0006548	218.1387	72.8	0	pos
Linoleamide	C18H33NO	6.7404	HMDB0062656	280.2634	38.8	0	pos
3-Methyl-2-Oxovaleric Acid	C6H10O3	4.2876	HMDB0000491	129.0549	0	90.2	neg
Gonal	C18H28O	6.5168	HMDB0252913	278.2478	63.6	0	pos
Phenylacetylglycine	C10H11NO3	3.9882	HMDB0000821	194.0813	0	47.8	pos
Furanofukinin	C16H24O2	6.6499	HMDB0036640	293.1773	63.4	0	neg

### 3.7 The relationship between the metabolites and meat quality-related parameters

**Figure 2:**
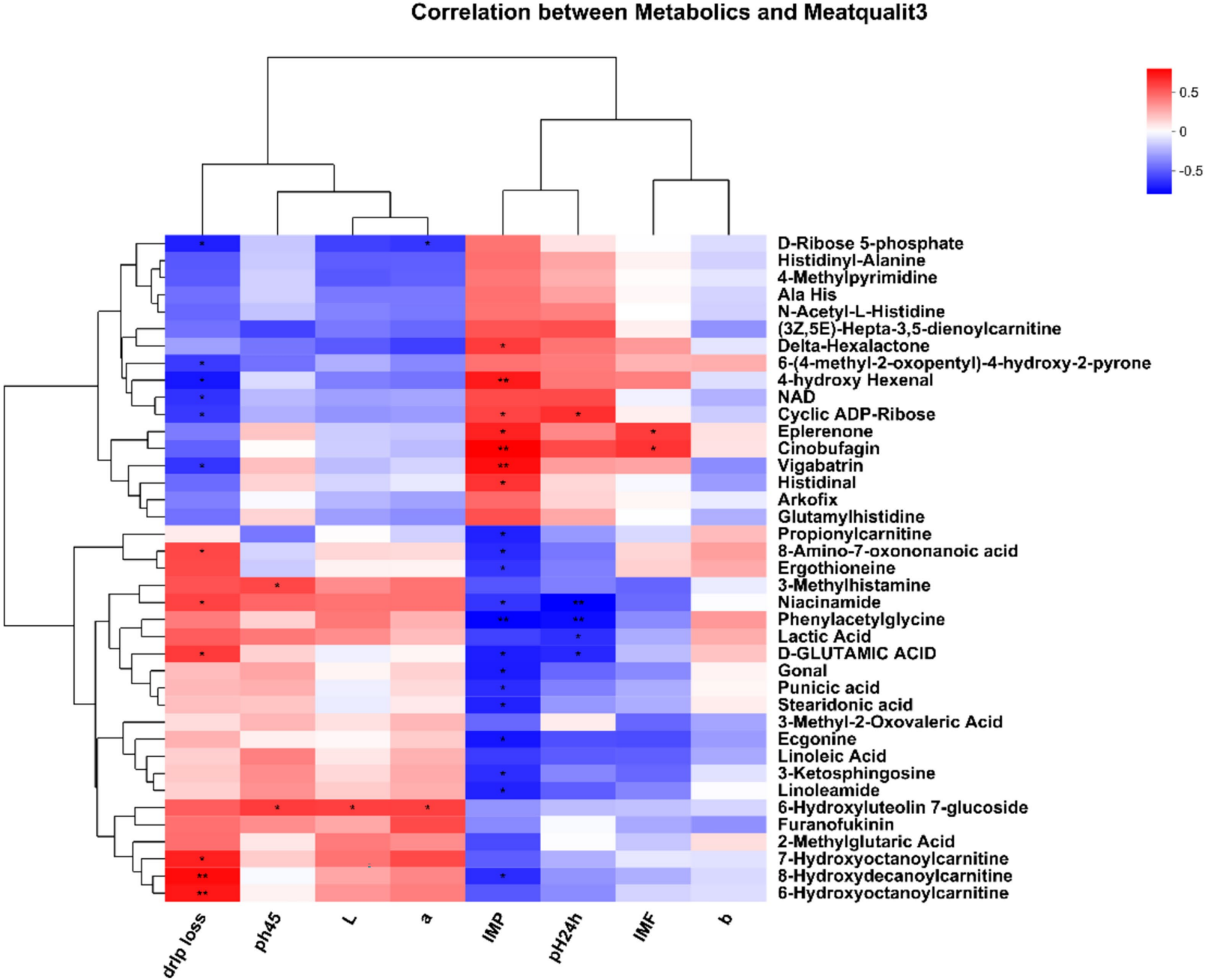
The correlation matrix between the metabolites and meat quality-related parameters. The heat-map encodes Pearson correlation coefficients (red = positive, blue = negative). *Indicates a significant difference in metabolite content (*p* ≤ 0.05), while **Denote an extremely significant difference (*p* ≤ 0.01). Both rows (metabolites) and columns (meat-quality parameters) were ordered by two-way hierarchical clustering using complete linkage and Euclidean distance to reveal co-regulated patterns.

As shown in Figure 2, the drip loss was positively associated with the content of D-glutamic acid, 8-amino-7-oxononanoic acid, niacinamide, 8-hydroxydecanoylcarnitine, 7-hydroxyoctanoylcarnitine, and 6-hydroxyoctanoylcarnitine, and was negatively associated with the content of 4-hydroxy hexenal, 6-(4-methyl-2-oxopentyl)-4-hydroxy-2-pyrone, D-ribose 5-phosphate, NAD+, cyclic ADP-ribose, and vigabatrin (*p* < 0.05). The IMF content was positively associated with the content of eplerenone and cinobufagin (*p* < 0.05). The IMP content was positively associated with the content of histidinal, cyclic ADP-ribose, vigabatrin, dclta-hcxalactonc, eplerenone, and cinobufagin, negatively associated with the content of 8-hydroxydecanoylcarnitine, propionylcarnitine, 8-amino-7-oxononanoic acid, ergothioneine, punicic acid, stearidonic acid, 3-ketosphingosine, gonal, linoleamide, ecgoninc, niacinamide, phenylacetylglycine, and D-glutamic acid (*p* < 0.05). The pH_24h_ was positively associated with the cyclic ADP-ribose and negatively associated with niacinamide, phenylacetylglycine, D-glutamic acid, and lactic acid (*p* < 0.05).

## 4 Discussion

The growth performance of animals can be reflected by the ADG, ADFI, and FCR. Chen et al. (23) demonstrated that adding 0.10% CGA to the diet of weaned piglets significantly increased the ADG and FCR. Li et al. (15) showed that 0.10% CGA supplementation also increased the growth performance of finishing pigs. Xie et al. (12) also suggested that dietary 0.01% CGA increased the final BW and ADG of finishing pigs. Similar to the above results, our study also found that the addition of 0.10% CGA showed beneficial trends in the final BW of Shaziling pigs, although without statistical significance. The growth-promoting effects might be associated with the beneficial effects of CGA on antioxidant capacity. Previous studies indicated that CGA could augment the activities of serum glutathione peroxidase, total superoxide dismutase, and catalase of pigs (23). However, one study showed the opposite results and found no significant impact of CGA supplementation (0.02, 0.04, and 0.08%) on ADG and FCR in finishing pigs. The reason for this incongruency is unclear but may be related to the differences in the sources, purity, and/or dosage of CGA.

The carcass traits refer to various characteristics related to the carcass of pigs after slaughter, such as the carcass yield, backfat thickness, loin-eye area, lean percentage, and fat percentage. These traits can reflect the rationality of diets and the production performance of animals (24). Xie et al. (12) found that the dressing percentage of finishing pigs was significantly increased by 0.05% CGA, without any significant effects on the carcass weight, carcass length, backfat depth, eye muscle area, and carcass lean percentage. Consistently, we also found that 0.10% CGA supplementation tended to increase the dressing percentage of Shaziling pigs, although the elevation did not reach to statistical significance. However, other carcass traits, such as carcass weight, carcass length, and backfat thickness, remained unaffected in response to CGA treatment. The higher the dressing percentage, the more the marketable carcass weight, and the stronger the purchasing willingness of slaughtering companies. Contrary to these findings, Wang et al. (25) reported that 0.04% CGA significantly increased the fat-free lean percentage and decreased the fat deposition of finishing pigs, without significant effects on the dressing percentage. There is no clear explanation for these divergent results, but the differences in experimental models and the varied doses used in each experiment are likely to be important issues. That being said, this study provided a strong hint about the beneficial effects of CGA-supplemented diets on the dressing percentage of Shaziling pigs, which is especially important in view of the potential for future large-scale application.

The serum biochemical parameters can reflect the nutrients’ metabolic status to different degrees. The TP content reflects the intake, absorption, and utilization of protein. The higher the TP content, the better the animal’s growth and development, and the lower the FCR (26). As a nutrient carrier, ALB helps maintain plasma osmotic pressure and acid–base equilibrium, aids in tissue repair, and provides nutrients (27). As a key indicator of nitrogen metabolism, UN reflects the status of protein utilization efficiency and the balance of dietary amino acids (28). The lower the serum UN content, the higher the nitrogen utilization efficiency, and the more amino acids and other raw materials for protein deposition (29, 30). In the current study, we found that TP and ALB levels increased, while UN levels decreased in the CGA group, which was mostly consistent with previous studies (25). A reduction in serum UN content is usually accompanied by an increase in muscle protein content (31, 32). Therefore, CGA may enhance nitrogen metabolism, and promote the nutrients absorption and deposition in meat products of Shaziling pigs.

Meat quality is extensively under the focus of intensive research in animal industry, and is generally reflected by parameters like pH, drip loss, meat color, IMF content, and shear force (18). Muscle pH is a direct factor influencing water-holding capacity, meat color, and tenderness, and can reflect the rate of muscle glycolysis after slaughter (33). The slower the rate of glycolysis, the less lactic acid is produced, the higher the pH value, the less water exudation loss (34). Drip loss reflects the water-holding capacity of muscle tissue (35), and has been demonstrated to be positively correlated with the lactate content (36). In the current study, after adding CGA, the decline rate of pH slowed down (as manifested by the increased pH_24h_), and the drip loss decreased, which suggested that CGA exerted positive effects on improving the meat quality of Shaziling pigs. These results are mostly consistent with previous findings, which already showed that dietary 0.05% CGA supplementation significantly increased the pH_24h_ in finishing pigs (12), and 0.10% CGA supplementation could significantly reduce the drip loss (15). In this study, the positive effects of CGA on pH_24h_ and drip loss of Shaziling pigs may be at least partially due to the decreased rate of glycolysis and the reduction of lactic acid content. Since metabolomic analyses of this study revealed that CGA supplementation significantly decreased the lactic acid content, which was negatively related to the pH_24h_. Interestingly, earlier studies confirm and extend the observation of this study by demonstrating that CGA decreases the production of lactic acid via inhibiting the oxidation of sarcoplasmic reticulum Ca^2+^-ATPase, thus augmenting the ultimate pH value of pork (15, 37). In addition, the IMF content is a key factor that affects the flavor, tenderness, and juiciness of meat. The higher the IMF content, the better the meat quality (38). In general, when the IMF content is 3.4% ~ 4.5%, the meat flavor is rich and fragrant (39). In the current study, the IMF content of the CGA group was 3.82%, which was within the recommended range and was 26.91% higher than that in the Con group. Similar results were obtained in finishing pigs, in which the IMF content was markedly increased by 0.01% CGA supplementation (12). Moreover, the increase in IMF content might be mainly contributed to by the upregulated mRNA level of acetylCoA carboxylase, which promoted the excess glucose taken up by the LDM to be converted into fatty acids and stored as fat (12, 40). While the CGA group exhibited a numerical reduction in cooking losses (CGA: 25.33% vs. Control: 23.68%), this difference did not reach statistical significance (*p* = 0.449). This lack of significance likely stems from the relatively small sample size and the inherent high variability of cooking loss measurements. Taken together, dietary CGA supplementation may not only improve meat quality but also increase the nutrient value of Shaziling pigs.

Apart from the abovementioned parameters, IMP is also a key substance responsible for the taste and flavor of meat, and it is approximately 50 times more effective than monosodium Glu in enhancing meat flavor (41). In this study, 0.10% CGA dietary supplementation resulted in a significant increase in the content of muscular IMP. This finding is consistent with a previous study (32), in which an increase in the IMP content in the muscles of finishing pigs fed with 0.08% dietary apple polyphenol supplementation was observed (CGA is one of the essential ingredients of apple polyphenols). However, some studies did not observe a similar elevation in the IMP content after CGA supplementation (12, 13). In contrast, dietary CGA supplementation reduced the content of bitter substances (such as inosine and hypoxanthine) generated by IMP metabolism (12). Bearing in mind the described effects of CGA on the IMP content and metabolism, it is posited that dietary CGA supplementation improved the meat flavor of Shaziling pigs by decreasing the IMP degradation and thus increasing the IMP content.

Apart from influencing the nutritional value of meat, amino acids are also key flavor compounds and their precursor substances in meat quality (42). For instance, Asp and Glu are the primary umami-enhancing amino acids, while amino acids such as Pro and Ser can impart sweetness. Thus, these amino acids are crucial in shaping the characteristic flavor of pork. Moreover, amino acids can undergo the Maillard reaction with reducing sugars, a fundamental process in the development of meat’s distinctive flavors. Asp and Glu could react with reducing sugars by the Maillard reaction to produce fragrance, and the amino acid Pro gives rise to typical bread, rice, and popcorn flavours (43). The results of this study showed that CGA supplementation increased the flavor-related amino acids such as Glu, Asp., Pro, Arg, Tyr, and Ser, which brought higher hierarchy, pure thickness, and better overall taste to meat. Moreover, the normal metabolism of the human body is closely related to the concentrations of certain amino acids. For instance, Arg can improve immunity through the increased secretion of endogenous substances (such as leukocytes and phagocytes) from the immune system. Tyr is involved in the synthesis of certain hormones and exerts key roles in protein synthesis and cell signaling (44). As a metabolic intermediate, Ser is involved in the synthesis of methionine, glycine, and cysteine, and mainly plays roles in lipid metabolism, the immune system, and the central nervous system (45). Additionally, P-ser, a Ser derivative, serves as an important nutrient for the brain and is an essential component of the cell membrane, and possesses antioxidant properties (46). Meanwhile, P-ser can help the development and repair of injured muscle (47). Anserine is thought to play an important role in the physiological function of muscles, especially in buffering pH changes, anti-oxidation, and anti-inflammation (48). This study found that CGA supplementation increased the content of Arg, Pro, Tyr, Ser, P-ser, and anserine in the LDM of Shaziling pigs, indicating that CGA could elevate the nutritional value of Shaziling pork and meet people’s nutritional needs.

The sensory characteristics and nutritional value of pork are also related to fatty acid composition. The finding of Zhang et al. (49) has shown that the SFA proportion is positively correlated with the IMF content. Our previous studies have shown that the MUFA (especially C18:1n9c) proportion is positively correlated with IMF or pH_24h_, whereas the PUFA (especially C18:2n6c) proportion is negatively related to IMF or pH_24h_ (19). In this experiment, dietary CGA supplementation did not significantly affect the SFA proportion, but significantly increased the proportions of MUFA and C18:1n9c, and decreased the proportions of PUFA and C18:2n6c and the ratio of PUFA to SFA. The alteration of fatty acid composition in the LDM caused by dietary CGA supplementation was mostly consistent with the IMF and pH_24h_ results, suggesting that CGA supplementation may improve the fatty acid composition in the LDM and enhance the meat flavor of Shaziling pigs. Additionally, the increase in IMF content caused by dietary CGA supplementation may be partially attributed to the increased levels of C18:1n9 and decreased levels of C18:2n6 in the LDM of Shaziling pigs. This is supported by the observation that as subcutaneous fat accumulates, C18:1n9 levels also rise, whereas C18:2n6 levels fall (50).

Apart from the effects on the sensory characteristics and nutritional value of pork, fatty acid composition in food can also affect human health, especially the occurrence and development of metabolic diseases (such as obesity and cardiovascular diseases) (51). It has been well documented that a high intake of SFAs contributes to the incidence of cardiovascular diseases (52). In contrast, MUFAs exert a wide range of health-protective effects, including protection against heart diseases, reduction of blood sugar levels, and regulation of blood lipids. For example, C18:1n9 can lower the levels of lipids, cholesterol, and LDL-C in the blood, and promote energy consumption (53). Similarly, PUFAs, especially n-3 PUFAs, also possess numerous benefits, such as anti-inflammatory properties. A diet with a lower n-6/n-3 PUFA ratio is believed to protect against cardiovascular diseases and to improve health (54, 55). In the current study, dietary CGA supplementation increased the contents of C18:1n9 and decreased the contents of n-6 PUFAs and the n-6/n-3 PUFA ratio in the LDM. These results indicated that dietary CGA supplementation may improve the edible health level of Shaziling pork.

Muscle metabolomics in domestic animals has been reported to help to account for meat quality traits (56). Hence, we compared the muscle metabolome between Shaziling pigs in the Con and CGA groups and analyzed the correlations between the top 40 metabolites and meat quality-related parameters. We found that compared to the Con group, the CGA group exhibited significantly higher concentrations of cyclic ADP-ribose, both of which were positively associated with IMP and pH_24h_ and negatively related to the drip loss. These results suggest that CGA may improve postmortem meat quality by modulating redox- and energy-related metabolism, which is consistent with previous evidence that dietary CGA enhances antioxidant capacity and increases muscle inosinic acid content (13). Mechanistically, the higher abundance of cyclic ADP-ribose may indicate altered intracellular Ca^2+^ signaling and postmortem glycolytic regulation, processes closely associated with ultimate pH and water-holding capacity, whereas the increaseexenal points to a shift in n-3 PUFA-derived lipid oxidation products; together, these changes are consistent with the higher IMP, higher pH24h, and lower drip loss observed in the CGA group (57). Therefore, it is assumed that muscular cyclic ADP-ribose might be involved in various pathways for improved meat quality of Shaziling pigs in response to CGA diets.

## 5 Conclusion

In summary, dietary 0.1% CGA supplementation improved meat quality in Shaziling pigs by increasing pH_24h_ value and the content of IMF and IMP without impairing growth performance or carcass characteristics. These effects were accompanied by improved the profiles of amino acids and fatty acids, rdecreased lactic acid, and increased cyclic ADP-ribose in muscle These metabolites were associated with meat-quality traits and may be involved in CGA-related changes in muscle metabolism.

## Data Availability

The metabolites data presented in the study are deposited in the XXX repository, accession number MTBLS2990. https://www.ebi.ac.uk/metabolights/MTBLS2990.
