# Supplementary material for: Dietary 0.10% chlorogenic acid improves the meat quality of Shaziling pigs
Source: Front Vet Sci. 2026 Jul 31;13:1846532. doi: 10.3389/fvets.2026.1846532 (PMC13474399; doi:10.3389/fvets.2026.1846532)
Supplement: Supplementary file 1 [file Table_1.docx]

**Table S1** Composition and nutrient levels of the diets for Shaziling pigs (air-dried basis, %)

| Ingredients, % | Contents, % | |
| --- | --- | --- |
|  | Control | CGA |
| Corn | 69.45 | 71.40 |
| Soybean meal | 14.50 | 12.10 |
| Wheat bran | 13.00 | 12.45 |
| Glutamate | 0.00 | 1.00 |
| CaHPO_4_ | 0.73 | 0.73 |
| Stone powder | 1.02 | 1.02 |
| Salt | 0.30 | 0.30 |
| Premix^1^ | 1.00 | 1.00 |
| Nutrient levels^2^ |  |  |
| Digestible energy, MJ/kg | 13.31 | 13.20 |
| Crude protein | 13.27 | 13.14 |
| Ether extract | 1.96 | 1.87 |
| Crude fibre | 3.20 | 3.09 |
| Calcium | 0.63 | 0.61 |
| Total phosphorus | 0.51 | 0.49 |
| Available phosphorus | 0.25 | 0.25 |
| Glutamate | 2.42 | 3.25 |

^1^Premix supplied per kilogram of feed: 2200 IU Vitamin VA, 220 IU Vitamin D3, 0.5 mg Vitamin K3, 17.5 μg Vitamin B12, 3.5 mg riboflavin, 30 mg niacin, 10 mg d-pantothenic acid, 0.05 mg biotin, 0.3 mg folic acid, 1.0 mg thiamine, 7 mg pyridoxine, and 4.0 mg ethoxyquin, 150 mg Fe, 100 mg Zn, 30 mg Mn, 25 mg Cu, 0.5 mg I, 0.3 mg Co, and 0.3 mg Se.

^2^The crude protein of diet was measured according to GB/T 6432-2018 by using continuous flow analyzer (Auto Analyzer 3-AA3, Seal Analytic, American); The ether extract was measured according to GB/T 6433-2006 by using automatic fat analyzer (SOX416, Gerhardt, Germany); Crude fibre was measured according to the GB/T 6434-2022 by using automatic fiber analyzer (FT12, Gerhardt, Germany) The total phosphorus and calcium were measured according to T/SATA 028-2022 by using Agilent 5100 ICP-OES (5100, Agilent, American); the glutamate content was measured according to GB/T 18246-2019 by using a Amino Acid Analyzer L-8900 (L8900, Hitachi, Japan). Disgestible energy and available phosphorus were calculated according to the China Feed Composition and Nutritional Value Table (Edition 32, 2021).
